# Bacterial Communities Associated with *Porites* White Patch Syndrome (PWPS) on Three Western Indian Ocean (WIO) Coral Reefs

**DOI:** 10.1371/journal.pone.0083746

**Published:** 2013-12-31

**Authors:** Mathieu G. Séré, Pablo Tortosa, Pascale Chabanet, Jean Turquet, Jean-Pascal Quod, Michael H. Schleyer

**Affiliations:** 1 Agence pour la Recherche et la Valorisation Marines (ARVAM), Ste Clotilde, Réunion Island, France; 2 Oceanographic Research Institute (ORI), Durban, KwaZulu-Natal, South Africa; 3 Institut de la Recherche pour le développement (IRD), Ste Clotilde, Réunion Island, France; 4 Centre de Recherche et de Veille sur les maladies émergentes dans l'Océan Indien (CRVOI), Ste Clotilde, Réunion Island, France; 5 University of Réunion Island, Ste Clotilde, Réunion Island, France; King Abdullah University of Science and Technology, Saudi Arabia

## Abstract

The scleractinian coral *Porites lutea*, an important reef-building coral on western Indian Ocean reefs (WIO), is affected by a newly-reported white syndrome (WS) the *Porites* white patch syndrome (PWPS). Histopathology and culture-independent molecular techniques were used to characterise the microbial communities associated with this emerging disease. Microscopy showed extensive tissue fragmentation generally associated with ovoid basophilic bodies resembling bacterial aggregates. Results of 16S rRNA sequence analysis revealed a high variability between bacterial communities associated with PWPS-infected and healthy tissues in *P. lutea*, a pattern previously reported in other coral diseases such as black band disease (BBD), white band disease (WBD) and white plague diseases (WPD). Furthermore, substantial variations in bacterial communities were observed at the different sampling locations, suggesting that there is no strong bacterial association in *Porites lutea* on WIO reefs. Several sequences affiliated with potential pathogens belonging to the *Vibrionaceae* and *Rhodobacteraceae* were identified, mainly in PWPS-infected coral tissues. Among them, only two ribotypes affiliated to *Shimi*a *marina* (NR043300.1) and *Vibrio hepatarius* (NR025575.1) were consistently found in diseased tissues from the three geographically distant sampling localities. The role of these bacterial species in PWPS needs to be tested experimentally.

## Introduction

The scleractinian *Porites lutea*, commonly found on back reefs, lagoon and fringing reefs [1], is an important reef-building coral in the western Indian Ocean (WIO) reefs. Despite its widespread distribution, this hermatypic coral has shown a particular susceptibility to natural pressures such as predation [2], [3] and infestation by parasites [4], [5]. Moreover, it seems to be more vulnerable to infectious disease than many other coral species [6].

Of the 30 coral diseases described to date [7], [8], eight are known to affect *P. lutea* worldwide. On Indo-Pacific reefs, colonies of *P. lutea* have been recorded with signs of black band disease (BBD), white plaque syndrome (WPL), growth anomalies (GA), yellow band disease (YBD) and pink line syndrome (PLS) [8]. Surveys conducted in the Gulf of Kutch [9], Papua New Guinea [10] and Philippines [11] have recorded BBD outbreaks in this scleractinian coral. In addition, a study performed on coral health and diseases in the northern Egyptian Red Sea has revealed two other syndromes: *Porites* ulcerative white spot (PUWS) and a white syndrome (WS) so far unreported on *P. lutea* [12]. More recently, a white syndrome (WS) named *Porites* white patch syndrome (PWPS) was described on massive colonies of *P. lutea* on Western Indian Ocean (WIO) reefs [13]. This syndrome was characterised by diffuse, medium to large (50–300 mm diameter), circular to oblong tissue loss, surrounded by swollen white tissue. The dead skeleton was progressively colonised by opportunistic algae and *Cyanobacteria* [13].

To date, nothing is known about the aetiology of PWPS. Previous studies on other white syndromes (white band disease (WBD), white plague disease (WP), progressive white syndromes (PWS), Australian subtropical white syndrome (ASWS), *Acropora* white syndrome in American Samoa (AWS), and *Porites* bleaching with tissue loss (PBTL)) have characterized organisms (bacteria, ciliates, helminths, fungi, algae) associated with both healthy and diseased coral colonies. These investigations have allowed identification of a number of putative pathogens [14]–[22]. Evidence of the involvement of bacteria as causative agents has been suggested in some studies on several of the WS observed on scleractinian corals [23]–[26]. For example *Serratia marcescens* has been reported to be linked with white pox disease (WPD) in the Elkhorn coral *Acropora palmata* [25] and *Vibrio owensii* to be the aetiological agent of *Montipora* white syndrome (MWS) in the Hawaiian coral *Montipora capilata* [27]. However, some of these potential causative agents have i) not been fully characterised in terms of fulfilling all Koch’s postulates [28] or ii) have been biased by the execution of infection trials with a specific pathogen rather than testing for multiple potential pathogens. Finally, no clear link between the proposed pathogens and signs of disease has been demonstrated at the gross and cellular level in aquaria or the field.

This study aimed to provide the first characterisation of bacterial communities associated with healthy and PWPS-affected massive colonies of *P. lutea* in three WIO regions: Mayotte, South Africa and Reunion. The sampling sites included a continental African reef and two oceanic islands separated by over 1500 km of mostly oceanic water masses. These sites were selected to highlight general microbial patterns associated with this disease and the host. The investigation presented here used both histopathology and culture-independent molecular (clone libraries) techniques. The main objectives were to i) describe the microscopic morphology of lesions in corals, and ii) identify the most represented bacteria associated with both healthy and infected coral tissues collected at the three localities.

## Methods

The sampling of *Porites lutea* colonies for this study was authorised by the French Department of Ecology, Sustainable Development, Transportation and Housing (DEAL), the Isimangaliso Wetland Park (South Africa) and CITES (Permit no. FR1197400391-FR1197400394-1).

### Sample collection

**Figure 1:**
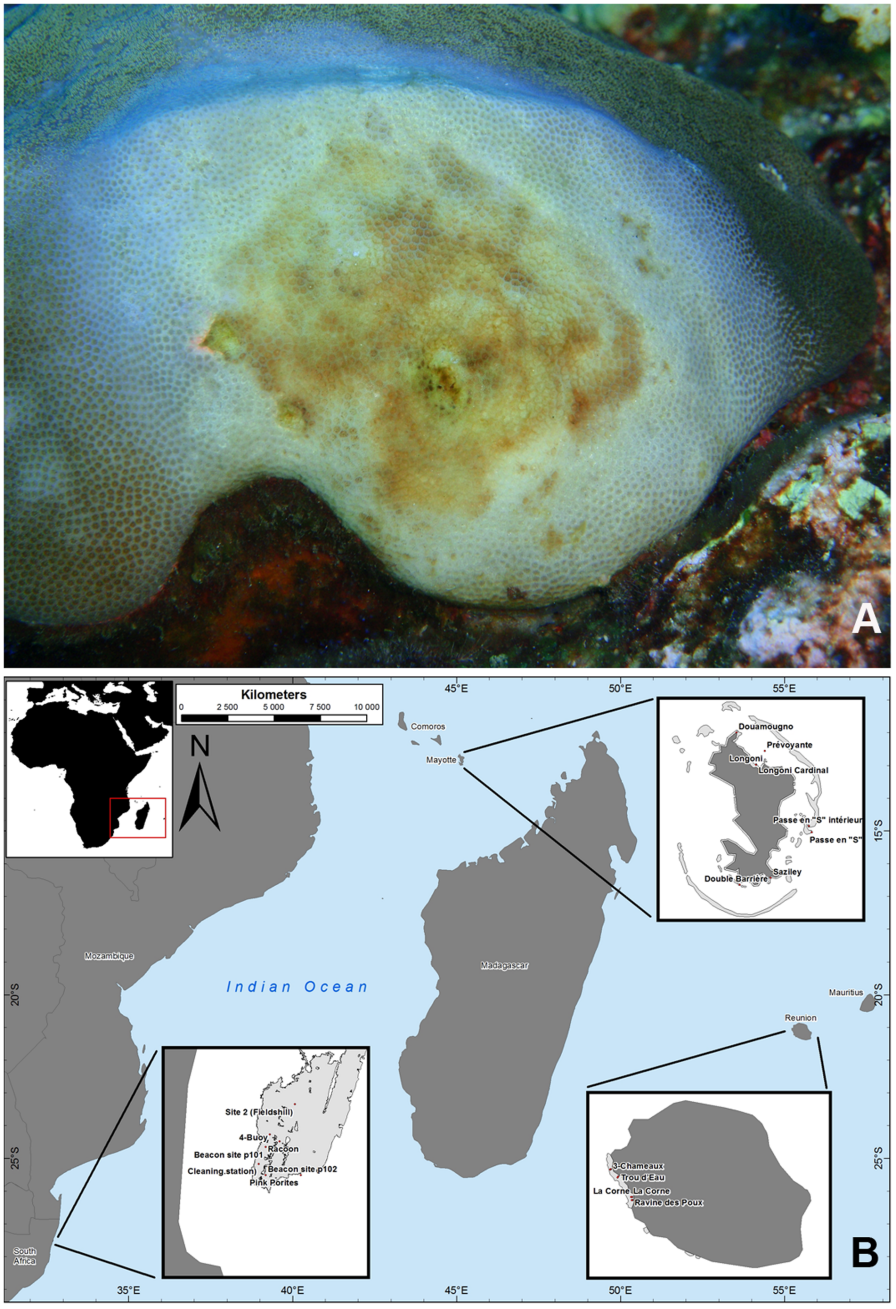
PWPS on *Porites lutea* (A) and map of the Western Indian Ocean showing the sampling locations (B).

Individual samples of *Porites lutea* exhibiting signs of PWPS were collected in South Africa, Reunion and Mayotte (Fig.1) using SCUBA. At each location, samples were collected from three healthy corals and colonies with signs of PWPS-infection (n_total_ = 18 samples). Diseased tissues (DT) were sampled from the lesion boundary interface with visually healthy tissue (HT), and samples of HT were collected from visually healthy colonies with no signs of disease. Cores of both HT and DT (2.2 cm diameter to a depth of 0.5–1 cm) were collected using a sterile stainless steel core tube and placed individually in sterile disposable 50 ml polypropylene centrifuge tubes. Upon reaching the shore, the seawater within each tube was drained for 10 minutes, replaced with 100% ethanol and stored at –20°C for subsequent DNA extraction and molecular analysis. Due to time and environmental constraints, corals exhibiting signs of PWPS could not be monitored for lesion progression.

Additional core samples were simultaneously collected at each location from five HT and five DT colonies using a hammer and chisel and fixed in 4% formalin for histopathology. Samples were protected in 1.5% (w/v) agarose to retain the spatial integrity of the tissues and microbial communities. They were then decalcified using 1% HCl and EDTA renewed every 12 h until the process was complete. Tissues from the decalcified fragments were dehydrated in a series of ethanol baths, cleared with xylene and embedded in paraffin wax. Sections of 6–8 µm were cut using a microtome, mounted on glass slides and stained with Harris haematoxylin and eosin with phloxine B to diagnose tissue fragmentation, necrosis and the presence of invasive organisms. Serial sections of the affected cells or tissues were examined under a light microscope at different magnifications (×200, ×400, ×1000) and photographed using NIS Element software (Nikon©). The number of colonies sampled per region for histology is presented in Table 1.

**Table 1:** Number of samples collected for histopathology, sections analysed and diagnosis partitioned by tissue category and region.

	Reunion		South Africa		Mayotte	
**Tissue categories**	**HT**	**DT**	**HT**	**DT**	**HT**	**DT**
**Samples**	5	5	5	5	5	5
**Cross-sections/sample**	10	10	10	10	10	10
**Samples with Ciliata**	0	1	0	0	1	0
**Samples with endophytic algae**	0	5	0	5	0	5
**Samples with ** ***Cyanobacteria***	0	3	0	0	0	2
**Samples with Nematoda**	0	5	0	4	0	0
**Samples with bacterial aggregates**	0	4	0	2	1	3

HT  =  healthy tissue. DT  =  disease tissue.

### DNA extraction

Bacterial genomic DNA from both HT and DT were extracted using the NucleoSpin® Soil Kit (NucleoSpin Extract II, *Macherey-Nagel*, Düren, Germany). Refrigerated samples were dried at room temperature, after which approximately 150 mg of both tissue and skeleton were scraped from their surface using a sterile scalpel blade, placed in a 1.5 ml centrifuge tube with 700 µl of lysis buffer and crushed using a fresh disposable plastic rod. Samples were then placed in lysing matrix tubes for DNA extraction. The DNA was eluted with 50 µl sterile elution buffer, verified by electrophoresis in agarose gels (1.5% wt/vol) stained with GelRed™ (Biotium Inc., Hayward, California, USA) and finally stored at –20°C.

### PCR Amplification

16S rRNA genes were amplified from DNA extracts by PCR using universal primers 28F and 1492R [29]. PCR reactions were carried out in a final volume of 25 µl containing GoTaq®Hot Start Green Master Mix (Promega, Madison, WI), 0.5 mM of each primer and 10 ng of template DNA. Reaction mixtures were incubated in a GeneAmp®PCR System 2700 thermal cycler. Amplification conditions for the PCR included an initial denaturing step of 4 min at 95.5°C, followed by 30 cycles at 94°C for 30 sec, 55°C for 60 sec and 7°C for 90 sec followed by a final extension step of 15 min at 72°C.

### Cloning and Sequencing

PCR was carried out using DNA templates prepared from each of the three individual DT or HT samples. PCR products were verified for quality, size and quantity by electrophoresis and spectrophotometry. Equimolar quantities of PCR products were then pooled for the three DT and HT samples at each locality (6 samples per locality) and each was subsequently separated by electrophoresis. Amplicons 1300–1500 bp long were excised from the gel under a UV transilluminator and the DNA was gel purified using NucleoSpin® Gel and a PCR Clean-up kit (*Macherey-Nagel*, Düren, Germany) for colony screening. The purified DNA was cloned into the pGEM-T vector system (Promega, Madison, WI) and ligation mixture was used to transform *Escherichia coli* JM109 competent cells. A total of 92–150 clones were randomly selected from each tissue category, spotted directly into 96-well plates and subjected to PCR with M13 forward and reverse primers (Inquaba Biotec™, Pretoria, South Africa). Amplification conditions for the PCR included an initial denaturing step at 94°C for 5 min followed by 35 cycles of 94°C for 1 min, 57°C for 1 min and 72°C for 1 min, with a final extension step at 72°C for 7 min. PCR products were checked for quality, size and quantity by electrophoresis in agarose gels (1.5% wt/vol) as described above and sent to GENOSCREEN (Campus de l’Institut Pasteur, Lille, France) for Sanger sequencing.

### Sequence analysis

The pair sequences obtained for each clone were edited and aligned using GENEIOUS™Pro (V.5.6.3) sequencing software. All high quality consensus sequences (HQ> 65%) were submitted to BLAST at the National Centre for Biotechnology Information (NCBI, www.ncbi.nlm.nih.gov) to determine their percentage similarity with known 16Sr DNA sequences. Sequences matching at a similarity level of 1) 97–100% were considered as belonging to the same species level, 2) 93–96% were considered as belonging to the same genus level and 3) <93% were considered to fall below the similarity of the genus level [14]. Bacterial rRNA sequences closely related to putative bacteria were aligned using Geneious alignment and rearranged manually. A phylogenetic tree was built using the Neighbor-Joining method of GENEIOUS™Pro (V.5.6.3). All 16r RNA gene sequences are accessible through the NCBI GeneBank database under accession numbers KF179641-KF180135.

### Statistical analysis

Multidimentional Scale (MDS) analysis of bacterial communities associated with healthy and PWPS-affected tissues of Porites lutea collected at Reunion (R), South Africa (RSA) and Mayotte (M) was performed using PRIMER (V.6.1.14). Data were square root-transformed and MDS analysis was carried out using the Bray–Curtis similarity coefficient. Finally, the Shannon-Weaver index (H) was calculated for each tissue category to characterise pooled bacterial diversities in healthy and diseased coral samples

## Results

### Microscopic morphology and spatial structure

**Figure 2:**
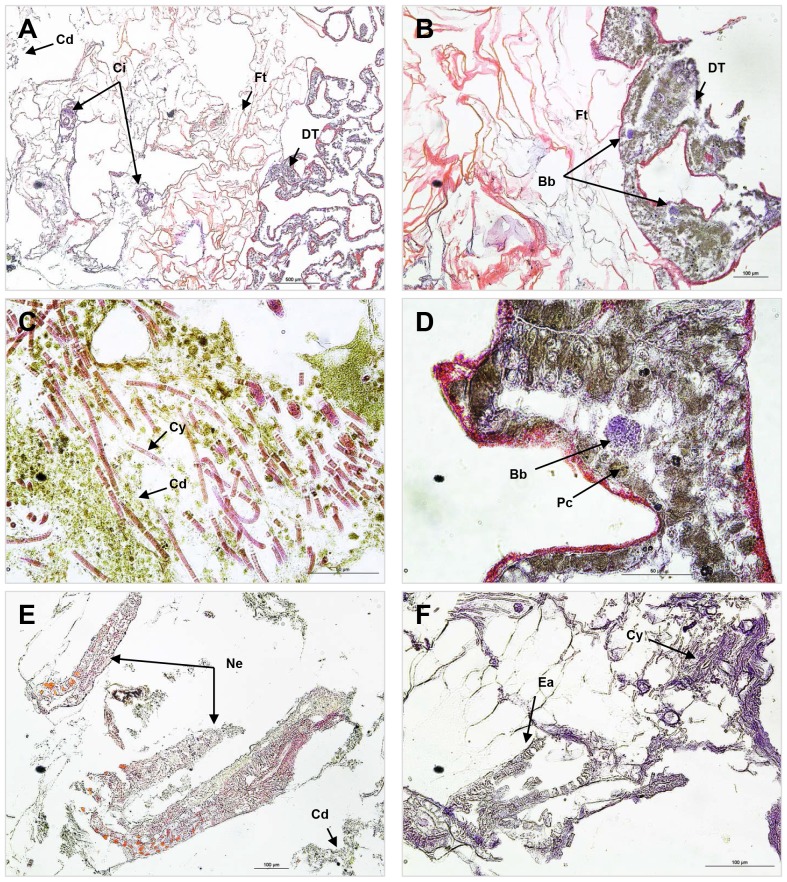
Photomicrographs of diseased *Porites lutea* coral tissues: *Porites* white patch syndrome (PWPS). **A**) Cross-section showing the well-defined boundary between fragmented (FT) and diseased tissue (DT); Cd  =  cell debris; Ci  =  Ciliata. **B**) *P. lutea* with PWPS. Note ovoid basophilic bodies (bb) like bacterial aggregates; Ft  =  fragmented tissue. **C**) Cross section of *P.lutea* affected by PWPS showing dead tissue full of cell debris (Cd) and *Cyanobacteria* (Cy). **D**) Close-up of an ovoid basophilic body (Bb). **E**) Nematoda (Ne) in the tissue debris. **F**) Endophytic algae (ea) in dead tissues.

Histological cross-sections of PWPS revealed extensive tissue breakdown and necrosis within the lesion area between the exposed skeleton and living tissue (Fig. 2A). Ovoid basophilic bodies resembling bacterial aggregates were visible within the mesogloea of the body wall, mainly in DT (Table 1), especially in the area of tissue fragmentation (Fig. 2B, D). These aggregates were seen in nine of the 15 samples collected from corals showing signs of PWPS at all three sampling locations. Among the 15 samples of HT, only one was found with such aggregates (Table 1). Other organisms, including *Cyanobacteria* (Fig. 2C, F), Nematoda (Fig. 2E), Ciliata (Fig. 2A) and algae (Fig. 2F) were also observed but only within dead tissue.

### Bacterial communities associated with healthy tissues

**Figure 3:**
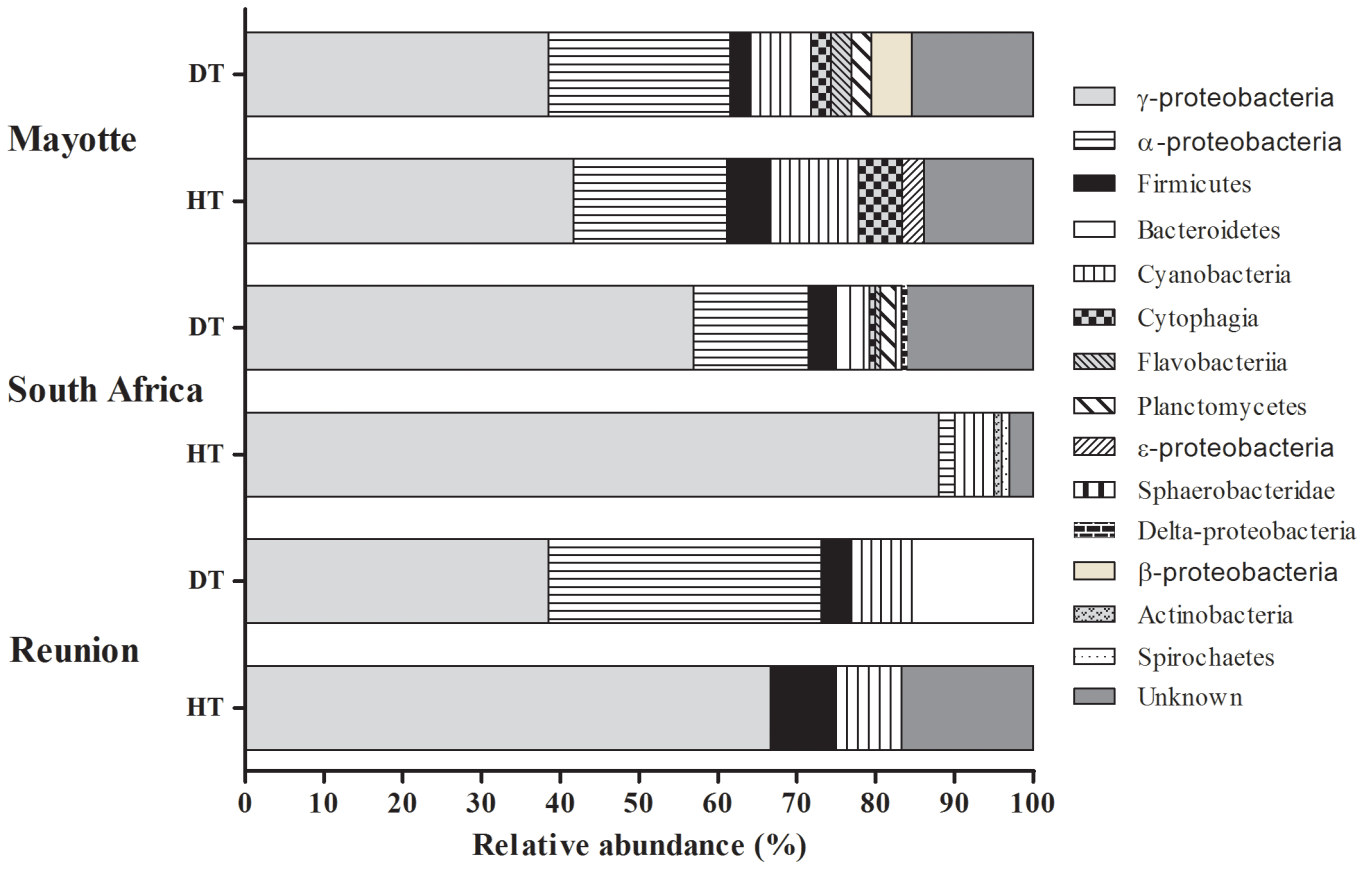
Relative abundance (%) of bacterial phyla retrieved from three diseased (DT) and three healthy (HT) samples of *Porites lutea* collected in Mayotte (MAY), South Africa (RSA) and Reunion (REU).

A total of 91, 74 and 100 16S rRNA sequences (818–1627 bp), subdivided into seven, six and four classes (Table 2 and Fig. 3) were obtained from healthy tissues collected in Mayotte (HT-M), South Africa (HT-RSA) and Reunion (HT-R) respectively (Table 2). Sequences retrieved from HT-M (Table 2 and Fig. 3) were mainly identified as members of the *γ-proteobacteria* (42.0%), *α-proteobacteria* (19.0%), *Cyanobacteria* (11.0%), *Firmicutes* (6.0%), *Cytophagia* (6.0%) and *?-proteobacteria* (3.0%). Of all sequences, 14.0% had no close relatives in the NCBI database and could only be classified as unknown bacterial clones. The bacterial diversity associated with HT-RSA (Table S1) was also dominated by sequences closely related to *γ-proteobacteria* (88.0%) followed by *Cyanobacteria* (5.0%), *α-proteobacteria* (2.0%), *Spirochaetes* (1.0%) and *Actinobacteria* (1.0%). HT-R (Table S1) seemed to contain less group diversity, mainly dominated by *γ-proteobacteria* (67.0%), *Cyanobacteria* (11.0%) and *Firmicutes* (6.0%). Unknown bacterial clones constituted 17.0% of all analysed sequences. The *γ-proteobacteria* retrieved from HT-M, HT-RSA and HT-R were dominated by bacterial species closely related to *Endozoicomonas elysicola* (accession no. NR041264), comprising 53.1%, 67.8% and 81.8% of the total *γ-proteobacteria* respectively. Sequences closely-related to *Vibrio fortis* (accession no. NR025575) were also common to bacterial communities associated with HT from all three sampling locations.

**Table 2:** Number of samples collected, clones retrieved by tissue category and region and diversity index (Shannon-Weaver).

	Reunion		South Africa		Mayotte	
	HT	DT	HT	DT	HT	DT
**Samples collected**	3	3	3	3	3	3
**Pooled samples**	1	1	1	1	1	1
**Random clones from pooled samples**	92	100	101	150	94	100
**Consensus sequences**	74	60	100	145	91	91
**Sequence lengths (bp)**	905–1498	904–1490	842–150	850–1498	1240–1483	855–1477
**Sequence quality (%)**	77.8–100	72.2–100	74.2–100	66.2–100	78.7–100	67.1–100
**Class/subdivision**	4	5	6	11	8	9
**Species**	12	26	21	53	36	39
**Diversity (Shannon-Weaver index)**	1.20	**2.83**	1.70	**3.37**	2.86	**3.29**

### Bacterial communities associated with diseased tissues

A total of 91, 145 and 60 16S rRNA gene clones (527–1602 bp), subdivided into 10, 12 and 5 classes (Table 2 and Fig. 3), was obtained from diseased tissues collected in Mayotte (PWPS-M), South Africa (PWPS-RSA) and Reunion (PWPS-R) respectively (Table S1). PWPS-M samples exhibited high diversity (Fig. 3), dominated by members of the *γ-proteobacteria* (38.0%) and *α-proteobacteria* (23.0%), followed by *Cyanobacteria* (6.0%), *Cytophagia* (6.0%), *Firmicutes* (3.0%), *Bacteroidetes* (3.0%), *β-proteobacteria* (3.0%), *Chloroplasts* (3.0%), *Planctomycetes* (3.0%) and *Flavobacteriia* (3.0%). Of the analysed sequences, 17.0% had no close relatives in the NCBI database and could only be classified as unknown bacterial clones. Similar trends emerged for PWPS-RSA (Fig. 3), the bacterial classes being dominated by *γ-proteobacteria* (56.9%) and *α-proteobacteria* (14.6%), followed by *Cyanobacteria* (3.5%), *Firmicutes* (3.5%), *Planctomycetes* (2.1%), *Bacteroidetes* (0.7%), *Cytophagia* (0.7%), *Delta-proteobacteria* (0.7%), *Flavobacteriia* (3.0%) and *Sphaerobacteridae* (3.0%). Again, some sequences (16.0%) had no close relatives in the NCBI database and could only be classified as unknown bacteria. PWPS-R (Table S1) samples were similarly dominated by members of the *γ-proteobacteria* (38.0%) and *α-proteobacteria* (23.0%), but contained only three other classes belonging to genera of *Bacteroidetes* (15.0%), *Cyanobacteria* (8.0%) and *Firmicutes* (4.0%).

Among the several bacterial classes found in this study, the *γ-proteobacteria Vibrio parahaemolyticus* (accession no. NR041838.1; n = 15), *V. fortis* (accession no. NR025575.1; n = 5) and *V. rotiferianus* (accession no. NR042081.1; n = 4), as well as the *α-proteobacteria Paracoccus yeei* (accession no. NR029038.1; n = 4), *Pseudoruegeria aquimaris* (accession no. NR043932; n = 2) and *Shimia marina* (accession no. NR043300.1; n = 3) were the best represented ribotypes in PWPS-M. The predominant bacterial ribotypes in PWPS-R were the *γ-proteobacteria E. elysicola* (accession no. NR041264; n = 12), *Photobacterium damselae* (accession no. NR042975.1; n = 4) and *Photobacterium* sp. (accession no. HQ697926; n = 3). The next most abundant sequences were closely related to the *α-proteobacteria P. yeei* (accession no. NR029038.1; n = 5), *Ruegeria pomeroyi* (accession no. NR028727; n = 3), *S. marina* (accession no. NR043300.1; n = 3) and *Silicibacter lacuscaerulensis* (accession no. NR029197; n = 3). Among the bacterial strains retrieved from PWPS-RSA, the *γ-proteobacteria*, *E. elysicola* (accession no. NR041264; n = 41) and *Oceanospirullum beijerinckii* (accession no. NR040784; n = 5), the *α-proteobacterium S. marina* (accession no. NR043300.1; n = 3) and the *Cyanobacterium Prochlorococcus marinus* (accession no. NR028762; n = 5) were the most representative species.

### Comparison of bacterial communities in healthy and diseased tissues

**Figure 4:**
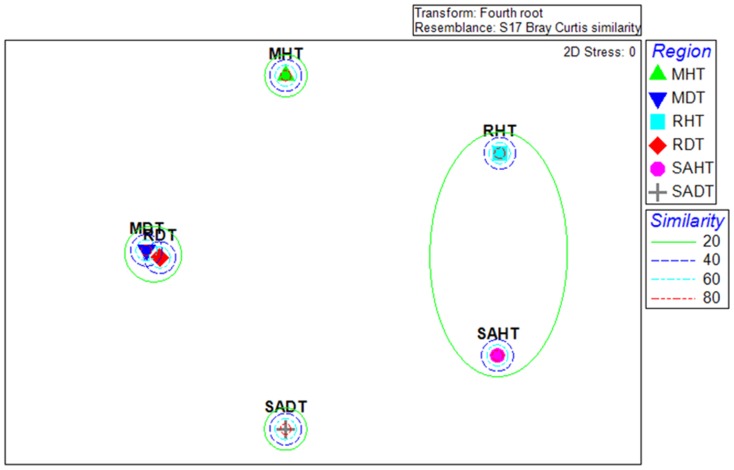
Multidimensional scaling (MDS) ordination of bacterial communities associated with healthy (HT) and PWPS-affected tissues (DT) of *Porites lutea* collected at Reunion (R), South Africa (RSA) and Mayotte (M).

Distinctly partitioned ribotypes were detected among diseased and healthy tissues samples. In total, 31 (77.8%), 54 (90.0%) and 24 (92.3%) bacterial ribotypes were exclusively associated with PWPS-M, PWPS-RSA and PWPS-R respectively, while 28 (77.8%), 17 (73.9%) and 10 (83.3%) were found only in HT-M, HT-RSA and HT-R respectively. Multidimentional scaling (MDS, Fig. 4B) analysis performed on the composition of bacterial 16S rRNA gene of each tissue categories revealed four distinct clusters representing four distinct bacterial communities. Similarities in bacterial composition in RDT and MDT were detected but SAHT, RHT, SADT and MHT samples exhibited more variability (Fig. 4). In addition, the bacterial diversity identified in PWPS tissues collected on the three WIO coral reefs was higher than in HT (Table 2). For instance, 39, 60 and 26 16S rRNA gene sequences affiliated to bacterial genera/species were identified in PWPS-M, PWPS-RSA and PWPS-R respectively, whereas only 36, 23 and 12 were obtained in HT-M, HT-RSA and HT-R respectively (Table 2). Among these, only six ribotypes were commonly detected in PWPS samples from the three sampling localities and were closely related to *P. yeei* (accession no. NR025491), *P. aquimaris* (accession no. NR029038.1), *S. marina* (accession no. NR043300.1), *V. fortis* (accession no. NR025575.1), *V. hepatarius* (accession no. NR025575.1) and *V. parahaemolyticus* (accession no. NR041838.1). In HT, only 16S rRNA gene sequences affiliated to bacterial species *E. elysicola* (accession no. NR041264) and *V. fortis* (accession no. NR025575.1) were common in samples collected at the three sampling localities.

## Discussion

### Histological observations

Corals exhibiting signs of PWPS revealed extensive tissue fragmentation, generally associated with ovoid basophilic bodies resembling bacterial aggregates within the mesoglea of the body wall. These aggregates were seen in 60% of all samples collected from corals with signs of PWPS. However, these aggregates could not be directly linked with the pathology as there was no clear evidence of inflammatory response or tissue lysis associated with these ovoid bodies. In addition, some clusters of basophilic bodies were also observed in sections of one healthy sample of *P. lutea*, preventing definitive conclusion that they constituted a bacterial infectious agent in the PWPS lesions. Similar observations on bacterial aggregates have been previously reported in several histopathological studies, in both healthy and WS-infected colonies of *Acropora* spp., *Pocillopora meandrina* and *Porites compressa* [22], [30]. Direct identification from formalin-fixed, paraffin-embedded coral tissue combined with descriptions of cellular changes over time may be a viable option to identify the role of these aggregates in PWPS. Other organisms, including *Cyanobacteria*, Nematoda, Ciliata and endophytic algae, were also observed on diseased tissues and were generally associated with the dead epidermis and cell debris. No obvious evidence of the direct physical ingress of these organisms into the tissue in cross-sections was observed suggesting that these were potentially opportunistic invaders.

### Variability in bacterial diversity in PWPS and HT

The bacterial communities in both healthy and PWPS-infected tissues of *P. lutea* were dominated at all three sampling locations by members of the *α-proteobacteria*, *γ-proteobacteria* and *Cyanobacteria* (Fig. 3). However, diseased corals exhibited higher bacterial diversity compared to healthy ones (Table 2). These results are consistent with recent studies which have reported lower bacterial abundance and diversity in healthy corals than those displaying signs of WPD [18], [19], [31] and WBD [14]. Examination of 16S rRNA gene sequences using cloning as a culture-independent molecular technique, revealed high variability between bacteria associated with PWPS-infected and healthy tissues of *Porites lutea*, with only a few ribotypes commonly found in both diseased and healthy tissues (Table S1). Among them, ribotypes similar to *Pseudoalteromonas* spp., *Paracoccus yeei* and *Amphritea balenae* have been previously identified in seawater [32], soil [33] and sediment [34] respectively, suggesting that these bacteria were present in the environment and opportunistically became resident in the coral mucus or associated with the healthy coral microbiota. Similar variations in the bacterial communities have been reported in several coral species affected by BBD [35], WPD [17], WBD in the Caribbean [14] and other WS in Australia and American Samoa [15], [20], [36]. The bacterial diversity found in PWPS was higher than in HT at all three localities. Our results were similar to those reported for white syndromes including *Acropora* white syndrome (AWS) on American Samoan reefs [20] and white plague disease in the Caribbean coral *Montastrea annularis* [17]. This difference in the composition of bacterial communities may suggest that disease agents impair the structure of natural bacterial communities. It is likely that compromised or dead tissues represent a “micro-niche” that can be colonised by more competitive and opportunistic bacteria in the surrounding water and sediments or transmitted by other marine organisms [14], [15], [17], [18], [37].

Interestingly, comparisons of bacterial communities associated with both PWPS-infected and healthy tissues also revealed distinct populations at the three sampling locations (Table S1, Fig. 4). This may suggest that no specific bacterial communities are associated with *P. lutea* on the WIO reefs. However some exceptions were recorded. For example, *E. elysicola* (accession no. NR041264) and *V. fortis* (accession no. NR025575.1) were found in both PWPS and HT collected at all the localities and seemed to be coral-specific. Another species, *V. rumoiensis* (accession no. NR024680), seemed to develop the same specific bacterial-coral association but was only found in HT sampled on South Africa and Mayotte reefs and not those at Reunion. These bacterial strains, apparently ubiquitous in HT, may play an important role in coral health and growth [14]. For instance the genus *Endozoicomonas*, found in several marine organisms [38]–[41], seems to play an important role in corals, notably in the biogeochemical cycling of sulphur [42]. *V. fortis* was initially described as a probiotic that out-competes pathogen strains [43], [44] or is involved in the recycling of dimethyl-sulfoniopropionate (DMSP), which may be detrimental to coral health [42]. However, further studies are needed to elucidate the ecological function of these genera in corals.

### Potential pathogens associated with PWPS

**Figure 5:**
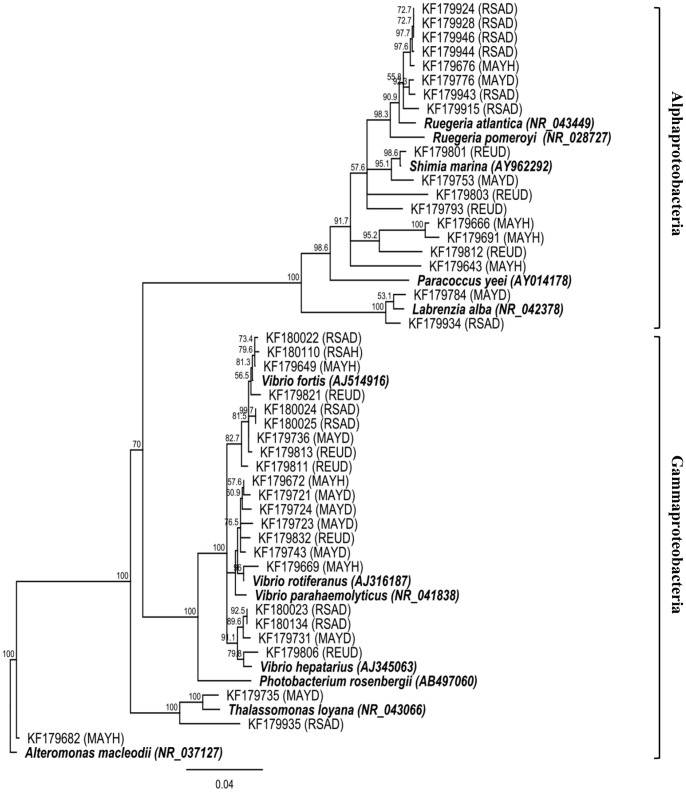
Neighbour-joining phylogenetic tree for the 16SrRNA gene sequences that were closely related to known and putative pathogens found in both healthy (HT) and *Porites* white patch syndrome (PWPS)-infected tissues (DT) of *Porites lutea* from Mayotte (MAY), South African (RSA) and Reunion (REU) corals. Numbers at each node are bootstraps values (%) obtained after 1000 iterations.

In our study, several 16S rRNA gene sequences were closely related (97–100% similarity) to bacteria associated with coral diseases or known pathogens. Interestingly, one sequence was detected in PWPS from all three sampling localities but absent in healthy corals. Blast identification associated with phylogenetic analysis (Fig. 5) showed it to be closely related to the *γ-proteobacteria V. hepatarius* (accession no. NR025575.1), isolated for the first time from the healthy wild white shrimp *Litopenaeus vannamei* in Ecuador [43]. Other 16S rRNA gene sequences affiliated to the family *Vibrionaceae* were associated with PWPS-infected tissues (Fig. 5). For instance, *V. fortis* was detected at all three sampling locations. This bacterium was first isolated from various marine organisms and has been reported to be pathogenic in corals [45], fish and crustacea [46], and is associated with several coral diseases including yellow band disease (YBD) in *Montastrea faveolata* [47] and BBD in the Red sea [48]. However, sequences affiliated with this species were also found in HT from all three localities, making this a less likely candidate for PWPS pathogenesis. In addition, ribotypes similar to *V. parahaemolyticus*, known to induce disease in humans [49] and many aquatic organisms [50], were identified as well as *V rotiferanus* associated with YBD in several Caribbean and Indo-Pacific scleractinian species [51]. However, similar sequences were found in healthy coral tissues or were not represented in diseased tissues at all three localities. Finally, sequences affiliated to *Shimia marina* (accession no. NR043300.1) were recorded only in PWPS-infected corals at all three sampling localities. This *roseobacter* was previously reported in the coral *Turbinaria mesenterina* infected by ASWS [15] but no evidence of its pathogenicity has been established in previous studies. The potential pathogens related to the sequences obtained in this study thus need to be isolated, cultured and inoculated in laboratory corals to ascertain their ability to induce disease in corals.

### Conclusions

This is the first study characterising bacterial communities associated with healthy and PWPS-infected *Porites lutea* coral colonies on WIO coral reefs. Microscopy revealed the inclusion of basophilic bodies like bacterial aggregates in the coral epidermis within the lesion area. We established that the structure of the microbial communities is different between diseased and healthy coral tissues, and between localities, by cloning the 16S rRNA gene as a culture-independent molecular method. Furthermore, higher bacterial diversity was observed in PWPS-infected tissues. This shift may be explained by a perturbation of the natural bacterial communities associated with coral holobionts which are progressively replaced by a succession of opportunistic bacteria including potential pathogens. Since the bacterial diversity at each of the three sites was assessed by analysing pooled samples, additional replicates including seasonal monitoring is needed to confirm the heterogeneity of bacterial species associated with PWPS in the areas studied. Several bacterial ribotypes affiliated to potential putative pathogens were consistently found among the 16S rRNA sequences derived from the PWPS lesions, and absent and/or poorly represented in HT. Isolation, culture and subsequent infection trials to satisfy Henle-Koch’s postulates would be needed to prove their pathogenicity.

## Supporting Information

Table S1 Bacterial 16S rRNA gene sequences from samples of apparently healthy (HT) and PWPS-diseased (DT) *Porites lutea* tissues collected at Mayotte (M), Reunion (R) and South Africa (SA).
(DOCX)
