# Supplementary material for: Bacterial Communities Associated with Porites White Patch Syndrome (PWPS) on Three Western Indian Ocean (WIO) Coral Reefs
Source: PLoS One. 2013 Dec 31;8(12):e83746. doi: 10.1371/journal.pone.0083746 (PMC3877091; doi:10.1371/journal.pone.0083746)
Supplement: Table S1 — Bacterial 16S rRNA gene sequences from samples of apparently healthy (HT) and PWPS-diseased (DT) Porites lutea tissues collected at Mayotte (M), Reunion (R) and South Africa (SA). (DOCX) [file pone.0083746.s001.docx]

Table S1. Bacterial 16S rRNA gene sequences from samples of apparently healthy (HT) and PWPS-diseased (DT) *Porites lutea* tissues collected at Mayotte (M), Reunion (R) and South Africa (SA).

| **Group/Subdivision affiliation** | **Closest genus/species in GenBank database** | **Best match (%)** | **GenBank Acc. No** | **M-HT** | **M-DT** | **R-HT** | **R-DT** | **SA-HT** | **SA-DT** |
| --- | --- | --- | --- | --- | --- | --- | --- | --- | --- |
| **Actinobacteria** | *Kineosporia rhamnosa* | 97 | NR028600 |  |  |  |  | **+** |  |
| **α-proteobacteria** | α-proteobacterium | 99 | AB571945 |  | **+** |  |  |  |  |
|  | *Henriciella marina* | 96 | NR044345 |  |  |  |  |  | **+** |
|  | *Erythrobacter aquimaris* | 99 | NR025789 |  |  |  |  |  | **+** |
|  | *Erythrobacter vulgaris* | 99 | NR043136.1 | **+** |  |  |  |  |  |
|  | *Hyphomonas adhaerens* | 95 | NR024937 | **+** |  |  |  |  |  |
|  | *Kiloniella laminariae* | 94 | NR042646.1 |  |  |  | **+** |  |  |
|  | *Labrenzia alba* | 98 | NR043040 |  | **+** |  |  |  | **+** |
|  | *Labrenzia marina* | 99 | NR043040 |  |  |  |  |  | **+** |
|  | *Leisingera aquimarina* | 98 | NR042670 |  |  |  | **+** |  |  |
|  | *Loktanella koreensis* | 97 | NR043741 |  |  |  |  |  | **+** |
|  | *Loktanella maricola* | 95 | NR044163 |  |  |  |  |  | **+** |
|  | *Oceanicaulis alexandrii* | 96 | NR025456.1 |  |  |  | **+** |  |  |
|  | *Mesorhizobium albiziae* | 99 | NR043549 |  |  |  |  |  | **+** |
|  | *Methylobacterium salsuginis* | 94-96 | NR044038 |  |  |  |  | **+** |  |
|  | *Paracoccus yeei* | 97-99 | NR029038.1 | **+** | **+** |  | **+** |  | **+** |
|  | *Parvularcula lutaonensis* | 99 | NR044474.1 | **+** |  |  |  |  |  |
|  | *Pseudoruegeria aquimaris* | 97-98 | NR043932 |  | **+** |  | **+** |  | **+** |
|  | *Pseudovibrio denitrificans* | 98 | NR041040.1 | **+** |  |  |  |  |  |
|  | *Roseovarius aestuarii* | 97 | NR044424 |  | **+** |  |  |  |  |
|  | *Roseovarius crassostreae* | 96 | NR041731 |  |  |  |  |  | **+** |
|  | *Ruegeria pomeroyi* | 98 | NR028727 |  |  |  | **+** |  | **+** |
|  | *Ruegeria atlantica* | 97 | NR043449 |  |  |  |  |  | **+** |
|  | *Shimia marina* | 98 | NR043300.1 |  | **+** |  | **+** |  | **+** |
|  | *Silicibacter lacuscaerulensis* | 98 | NR029197 |  |  |  | **+** |  | **+** |
|  | *Sphingomonas echinoides* | 99 | NR024700.1 |  |  |  | **+** |  |  |
|  | *Sphingopyxis flavimaris* | 100 | NR025814 |  | **+** |  |  |  |  |
|  | α-proteobacterium | 94 | JQ579969.1 | **+** |  |  |  |  |  |
|  | Rhodospirillales sp. | 95 | HM798908.1 | **+** |  |  |  |  |  |
|  | *Roseobacter* sp. | 99 | EF092256 |  | **+** |  |  |  |  |
|  | *Thalassobius aestuarii* | 98 | NR042903 |  |  |  |  |  | **+** |
|  | *Thalassobius gelatinovorusc* | 98 | NR043447 |  |  |  |  |  | **+** |
|  | *Thalassobius* sp. | 99 | FJ403051 |  | **+** |  |  |  |  |
| **Bacteroidetes** | *Bacteroidetes* sp. | 96 | HM593523 |  |  |  |  |  | **+** |
|  | Flavobacteria sp. | 95 | AM279213 |  | **+** |  |  |  |  |
|  | *Fabibacte*r sp. | 98 | HQ270264.1 |  |  |  | **+** |  |  |
|  | *Flexibacter elegans* | 100 | NR040908.1 |  |  |  | **+** |  |  |
|  | *Lewinella nigricans* | 98 | NR028695 |  |  |  | **+** |  |  |
|  | *Marinoscillum furvescens* | 100 | NR040920.1 |  |  |  | **+** |  |  |
| **β-proteobacteria** | *Delftia tsuruhatensis* | 99 | NR024786.1 |  | **+** |  |  |  |  |
| **Chloroplast** | Uncultured organism | 97 | GU119563.1 |  | **+** |  |  |  |  |
| **Cyanobacteria** | *Halospirulina tapeticola* | 92 | NR026510 |  |  |  | **+** |  | **+** |
|  | *Halospirulina tapeticola* | 92 | NR026510.1 |  |  | **+** |  |  |  |
|  | *Limnothrix* sp. | 96 | DQ889938.1 | **+** |  |  |  |  |  |
|  | *Planktothricoides raciborskii* | 92 | NR040858 |  |  |  |  |  | **+** |
|  | *Prochlorococcus marinus* | 97 | NR028762 | **+** | **+** |  |  |  | **+** |
|  | *Pseudophormidium* sp. | 100 | AB512143.1 | **+** |  |  |  |  |  |
|  | Uncultured cyanobacterium | 99 | HM474900.1 | **+** |  |  |  |  |  |
|  | Uncultured cyanobacterium | 99 | HQ242399.1 |  | **+** |  |  |  |  |
|  | Uncultured cyanobacterium | 94 | FJ516952.1 |  |  |  | **+** |  |  |
| **Cytophagia** | *Flammeovirga* sp. | 98 | NR041394.1 | **+** |  |  |  |  |  |
|  | *Flammeovirga* sp. | 98 | AB681285.1 |  | **+** |  |  |  |  |
|  | Flexibacteraceae sp. | 91 | FJ425608.1 | **+** |  |  |  |  |  |
|  | *Marinoscillum furvescens* | 94 | NR040920 |  |  |  |  |  | **+** |

Table S1. Continued

| **Group/Subdivision affiliation** | **Closest genus/species in GenBank database** | **Best**  **match (%)** | **GenBank Acc. No** | **MHT** | **MDT** | **RHT** | **RDT** | **SAHT** | **SADT** |
| --- | --- | --- | --- | --- | --- | --- | --- | --- | --- |
| **ɛproteobacteria** | *Arcobacter* sp. | 97 | DQ917897.1 | **+** |  |  |  |  |  |
| **λ-proteobacteria** | λ-proteobacterium clone | 99 | HM593548 |  |  |  |  |  | **+** |
| **Firmicutes** | *Alkaliphilus crotonatoxidans* | 100 | NR041892 |  |  |  | **+** |  |  |
|  | *Bacteroides capillosus* | 100 | NR025670.1 | **+** |  |  |  |  |  |
|  | *Clostridium clariflavum* | 100 | NR041235 |  |  |  |  |  | **+** |
|  | *Cryptanaerobacter phenolicus* | 94 | NR025757.1 |  |  | **+** |  |  |  |
|  | *Epulopiscium* sp. | 95-96 | DQ917864 |  | **+** |  |  |  |  |
|  | *Gemella haemolysans* | 100 | NR025903 |  |  |  |  |  | **+** |
|  | *Proteiniborus ethanoligenes* | 97 | HM585026.1 | **+** |  |  |  |  |  |
|  | *Sporomusa malonica* | 100 | NR025416 |  |  |  |  |  | **+** |
| **Flavobacteriia** | *Flavobacterium* sp. | 95 | FJ745113 |  | **+** |  |  |  |  |
|  | *Gaetbulibacter marinus* | 97 | NR044090 |  |  |  |  |  | **+** |
| **γ-proteobacteria** | *Aeromonas bivalvium* | 94 | NR043885 |  |  |  |  | **+** |  |
|  | *Aeromonas hydrophila* | 92 | NR042155 |  |  |  |  | **+** |  |
|  | Alteromonadales sp. | 99 | FJ952789.1 |  | **+** |  |  |  |  |
|  | *Alteromonas genovensis* | 94 | NR042667.1 | **+** |  |  |  |  |  |
|  | *Alteromonas macleodii* | 99 | NR037127 | **+** | **+** |  | **+** |  |  |
|  | *Alteromonas* sp. | 99 | FJ952780.1 |  | **+** |  |  |  |  |
|  | *Amphritea atlantica* | 93-94 | NR042455 |  |  |  |  | **+** | **+** |
|  | *Amphritea balenae* | 98 | NR041617.1 |  |  |  | **+** |  |  |
|  | *Azorhizophilus paspali* | 93 | NR042070 |  |  |  |  | **+** | **+** |
|  | *Azotobacter beijerinckii* | 92 | NR042071 |  |  |  |  | **+** | **+** |
|  | *Dasania marina* | 93-94 | NR043175 |  |  |  |  |  | **+** |
|  | *Endozoicomonas*  *elysicola* | 92-99 | NR041264 | **+** |  | **+** | **+** | **+** | **+** |
|  | *Enterovibrio coralii* | 98 | NR042342.1 |  |  |  | **+** |  |  |
|  | *Ferrimonas balearica* | 94 | NR027602 |  |  |  |  |  | **+** |
|  | *Halomonas aquamarina* | 100 | NR042063 |  |  |  |  | **+** |  |
|  | *Marinobacter lutaoensis* | 93 | NR025116 |  |  |  |  | **+** |  |
|  | *Neptuniibacter caesariensis* | 97 | NR042749 |  | **+** |  | **+** |  |  |
|  | *Oceanospirillum beijerinckii* | 95-100 | NR040784 |  |  |  |  |  | **+** |
|  | *Photobacterium damselae* | 96-98 | NR042975.1 |  |  | **+** |  | **+** | **+** |
|  | *Photobacterium frigidiphilum* | 97 | NR042964 |  |  |  |  | **+** |  |
|  | *Photobacterium halotolerans* | 99 | NR042975 |  |  |  |  | **+** |  |
|  | *Photobacterium lutimaris* | 98-99 | NR043902 |  |  |  |  |  | **+** |
|  | *Photobacterium rosenbergii* | 99 | NR042343.1 | **+** |  |  |  |  | **+** |
|  | *Photobacterium* sp. | 97-98 | HQ697926 |  |  | **+** |  |  |  |
|  | *Pseudoalteromonas agarivorans* | 93 | NR025509 |  |  |  |  |  | **+** |
|  | *Pseudoalteromona haloplanktis* | 95 | NR044837 |  |  |  |  |  | **+** |
|  | *Pseudoalteromonas mariniglutinosa* | 98 | NR028992 | **+** | **+** | **+** | **+** |  |  |
|  | *Pseudoalteromonas phenolica* | 98-100 | NR028809 | **+** | **+** |  |  |  |  |
|  | *Pseudoalteromonas* sp. | 99 | AF343949.1 |  | **+** |  |  |  |  |
|  | *Pseudoalteromonas* sp. | 99 | FJ457155 | **+** |  |  |  |  |  |
|  | *Pseudoalteromonas* sp. | 99 | FJ170037.1 | **+** |  |  |  |  |  |
|  | *Pseudoalteromonas* sp. | 99 | HQ342691 | **+** |  |  |  |  |  |
|  | *Pseudoalteromonas* sp. | 99 | AB457045 | **+** |  |  |  |  |  |
|  | *Pseudomonas alcaliphila* | 93 | NR024734 |  |  |  |  | **+** |  |
|  | *Pseudomonas fragi* | 94 | NR024946.1 |  |  | **+** |  |  |  |
|  | *Pseudomonas indica* | 98 | NR028801 |  |  |  |  |  | **+** |
|  | *Pseudomonas lutea* | 92 | NR029103 |  |  |  |  |  | **+** |
|  | *Pseudomonas mosselii* | 91 | NR024924.1 | **+** |  |  |  |  |  |
|  | *Pseudomonas* sp. | 100 | AJ551160.1 |  |  |  | **+** |  |  |
|  | *Rhodanobacter lindaniclasticus* | 96 | NR024878 |  |  |  |  |  | **+** |
|  | *Thalassomonas loyana* | 98 | NR043066.1 |  | **+** |  |  |  | **+** |
|  | Alteromonadales sp. | 99 | FJ403097.1 |  | **+** |  |  |  |  |
|  | *Vibrio crassostreae* | 98 | NR044078 |  |  | **+** |  |  |  |

Table S1. Continued

| **Group/Subdivision affiliation** | **Closest genus/species in GenBank database** | **Best match (%)** | **GenBank Acc. No** | **MHT** | **MDT** | **RHT** | **RDT** | **SAHT** | **SADT** |
| --- | --- | --- | --- | --- | --- | --- | --- | --- | --- |
|  | *Vibrio fortis* | 98-99 | NR025575.1 | **+** | **+** | **+** | **+** | **+** | **+** |
|  | *Vibrio furnissii* | 99 | NR036790 |  |  |  |  |  | **+** |
|  | *Vibrio gallicus* | 100 | NR025740 |  |  |  |  |  | **+** |
|  | *Vibrio gazogenes* | 98 | NR029256 |  |  |  |  | **+** |  |
|  | *Vibrio harveyi* | 99 | NR043165.1 | **+** | **+** |  |  |  |  |
|  | *Vibrio hepatarius* | 99 | NR025491.1 |  | **+** |  | **+** |  | **+** |
|  | *Vibrio natriegens* | 97 | NR026124.1 |  |  | **+** |  |  |  |
|  | *Vibrio parahaemolyticus* | 98-99 | NR041838.1 | **+** | **+** |  | **+** |  |  |
|  | *Vibrio rotiferianus* | 99 | NR042081.1 |  | **+** |  | **+** |  |  |
|  | *Vibrio rumoiensis* | 98 | NR024680 | **+** |  |  |  | **+** |  |
|  | *Vibrio* sp. | 98 | AB470934 |  | **+** |  |  |  |  |
|  | *Zooshikella ganghwensis* | 93 | NR025668 |  |  |  |  | **+** | **+** |
| **Planctomycetes** | Planctomycetes | 95-97 | JF443763 |  | **+** |  |  |  |  |
|  | *Rhodopirellula baltica* | 100 | NR043384 |  |  |  |  |  | **+** |
|  | *Singulisphaera acidiphila* | 97 | NR042662 |  |  |  |  |  | **+** |
| **Sphaerobacteridae** | *Sphaerobacter thermophilus* | 100 | NR042118 |  |  |  |  |  | **+** |
| **Spirochaetes** | *Leptospira borgpetersenii* | 99 | JQ988862 |  |  |  |  | **+** |  |
| **Unknown** | Uncultured bacterium | 98-99 | EU636648 | **+** |  |  |  |  |  |
|  | Uncultured bacterium | 95 | GU119164.1 | **+** |  |  |  |  |  |
|  | Uncultured bacterium | 99 | GU118981.1 | **+** |  |  |  |  |  |
|  | Uncultured bacterium | 96 | FJ202586.1 | **+** |  |  |  |  |  |
|  | Uncultured bacterium | 96 | GU119041.1 | **+** |  |  |  |  |  |
|  | Uncultured bacterium | 99 | JQ347309.1 |  | **+** |  |  |  |  |
|  | Uncultured bacterium | 97 | HM768687 |  | **+** |  |  |  |  |
|  | Uncultured bacterium | 99 | FJ203318 |  | **+** |  |  |  |  |
|  | Uncultured bacterium | 99 | FJ202906.1 |  | **+** |  |  |  |  |
|  | Uncultured bacterium | 99 | FJ202762 |  | **+** |  |  |  |  |
|  | Uncultured bacterium | 98 | JF514283.1 |  | **+** |  |  |  |  |
|  | Uncultured bacterium | 97-98 | GU220747.1 |  |  | **+** |  |  |  |
|  | Uncultured bacterium | 92 | JF261520.1 |  |  | **+** |  |  |  |
|  | Uncultured bacterium | 98-99 | HM445412 | **+** |  |  |  |  |  |
|  | Uncultured bacterium | 98 | DQ200473 |  |  |  |  |  | **+** |
|  | Uncultured bacterium | 98 | JF272035 |  |  |  |  |  | **+** |
|  | Uncultured bacterium | 99 | GU293218 |  |  |  |  |  | **+** |
|  | Uncultured bacterium | 99 | GU472290 |  |  |  |  |  | **+** |
|  | Uncultured bacterium | 97-98 | FJ202885 |  |  |  |  |  | **+** |
|  | Uncultured bacterium | 98 | JF915116 |  |  |  |  |  | **+** |
|  | Uncultured bacterium | 97 | FJ952694 |  |  |  |  |  | **+** |
|  | Uncultured bacterium | 97-99 | FJ202885 |  |  |  |  |  | **+** |
|  | Uncultured bacterium | 96 | FJ203501 |  |  |  |  |  | **+** |
|  | Uncultured bacterium | 99 | FJ203506 |  |  |  |  |  | **+** |
|  | Uncultured bacterium | 99 | FJ202970 |  |  |  |  | **+** |  |
